# Knowledge mapping and bibliometric analysis of metabolic phenotypes in childhood and adolescent obesity: research hotspots and emerging trends from 2000 to 2026

**DOI:** 10.3389/fpubh.2026.1907434

**Published:** 2026-07-16

**Authors:** Guodong Ren, Zhanpeng Feng, Xiuzhi Wang, Jun He, Zhongtao Yu, Liquan Cao

**Affiliations:** 1 Tianjin University of Sport, Tianjin, China; 2 Tianjin Key Laboratory of Sports and Health Integration and Health Promotion, Tianjin, China; 3 Tianjin Sports Comprehensive Support Center, Tianjin Institute of Sports Sciences, Tianjin, China

**Keywords:** children and adolescents, metabolic phenotype, metabolically healthy obesity, metabolically unhealthy obesity, visualization analysis

## Abstract

**Background:**

Childhood and adolescent obesity is increasingly understood as a heterogeneous condition in which excess adiposity may be accompanied by distinct cardiometabolic phenotypes. Metabolically healthy obesity and metabolically unhealthy obesity have become important concepts for risk stratification, but the overall knowledge structure and emerging research directions in this field remain insufficiently mapped.

**Methods:**

Records on metabolic phenotypes in childhood and adolescent obesity published between 1 January 2000 and 20 February 2026 were retrieved from the Web of Science Core Collection and PubMed on 20 February 2026. English-language articles and reviews were independently screened by two researchers. After database merging, deduplication and data cleaning, 4,963 records were retained for bibliometric analysis, including 4,384 WoSCC-retained records and 579 PubMed-only records. CiteSpace 6.4. R1, VOSviewer 1.6.20, R 4.5.2 and bibliometrix 5.4.1 were used to analyze annual publication trends, country and institutional collaboration, author and reference co-citation, keyword co-occurrence, clustering and burst terms. Citation-dependent analyses were based on WoSCC-retained records with complete cited-reference metadata.

**Results:**

Publication output increased overall and showed three broad phases: an initial phase centered on BMI criteria, obesity prevalence and metabolic syndrome recognition; a rapid growth phase associated with insulin resistance, glucose-lipid abnormalities and metabolic heterogeneity; and a recent consolidation phase emphasizing phenotype transition, gut microbiota, non-alcoholic fatty liver disease, physical activity, cardiometabolic health and precision intervention. The United States, Spain, China and England were leading contributors, although cross-regional collaboration remained uneven. Co-cited references, author networks and keyword clusters indicated that the field has moved from weight-based classification toward integrated cardiometabolic risk identification, with emerging attention to central adiposity, visceral fat, microbiome-related mechanisms and phenotype-guided management.

**Conclusion:**

Research on metabolic phenotypes in childhood and adolescent obesity is shifting from BMI-centered description toward risk-stratified interpretation of metabolic heterogeneity. Future studies should harmonize pediatric phenotype definitions, strengthen multicenter longitudinal cohorts and integrate body composition, fat distribution, insulin sensitivity, liver fat, inflammatory markers, lifestyle exposure and gut microbiota to support early identification and precision prevention of cardiometabolic risk.

## 1 Introduction

Obesity is now widely recognized as a chronic and complex disease caused by excessive adiposity and capable of inducing multi-organ and multi-system dysfunction (1). In children and adolescents, obesity increases the risk of cardiovascular disease, type 2 diabetes, metabolic syndrome, obstructive sleep apnea and other adverse health outcomes (2–8). Global evidence indicates that the burden of pediatric overweight and obesity has continued to rise over recent decades and is projected to remain a major public health challenge (9–14). This expanding burden makes it increasingly important to move beyond body weight alone and to identify the metabolic risk profiles that distinguish children with similar adiposity but different health trajectories.

A growing body of evidence shows marked metabolic heterogeneity among individuals with obesity (15–20). Commonly used phenotypes include metabolically healthy obesity and metabolically unhealthy obesity (15–17, 21). Metabolically unhealthy obesity is characterized by obesity accompanied by cardiometabolic abnormalities such as insulin resistance, impaired glucose tolerance, dyslipidemia and hypertension (22–26). By contrast, children classified as having metabolically healthy obesity may have preserved glucose, blood pressure and lipid profiles, although this phenotype is not necessarily benign and may transition to a metabolically unhealthy state over time (27, 28).

Research on obesity metabolic phenotypes has expanded across exercise intervention, lifestyle management, metabolomics, gut microbiota, body composition and precision risk stratification (22, 29–39). However, although individual clinical studies, epidemiological investigations and narrative reviews have examined specific aspects of metabolic phenotypes in pediatric obesity, the global publication patterns, major contributors, collaborative relationships, intellectual structure and thematic evolution of this field have not been comprehensively mapped. Bibliometric and visualization methods provide a useful approach for identifying research trends, collaboration networks, co-cited knowledge bases and emerging hotspots in a rapidly developing interdisciplinary field (40–42).

Therefore, this study aimed to conduct a comprehensive bibliometric analysis of research on metabolic phenotypes in childhood and adolescent obesity from 2000 to 2026. Using the Web of Science Core Collection as the core bibliometric database and PubMed as a supplementary biomedical search source, we mapped global publication trends, key countries and institutions, journal distribution, co-cited references, author networks, keyword clusters and emerging research themes. By doing so, this study provides a structured knowledge map of the field and clarifies how pediatric obesity research is shifting from BMI-based description toward metabolic risk stratification, phenotype transition and precision prevention.

## 2 Materials and methods

### 2.1 Eligibility criteria and screening

The inclusion criteria were as follows: (1) studies clearly related to metabolic phenotypes in childhood and adolescent obesity; (2) records with complete bibliographic information; (3) publicly published articles or reviews; (4) English-language publications.

The exclusion criteria were reports: (1) meeting abstracts, commentaries, book chapters and other non-research documents; (2) incomplete or unavailable records; (3) duplicate publications; non-English records; (4) adult-only studies; (5) records not focused on obesity metabolic phenotypes.

### 2.2 Data sources and search strategy

This study adopted a dual-database retrieval strategy. Literature was searched in the Web of Science Core Collection (WoSCC) and PubMed on 20 February 2026, covering publications from 1 January 2000 to 20 February 2026. The WoSCC search included the Science Citation Index Expanded, Social Sciences Citation Index and Emerging Sources Citation Index. WoSCC was selected as the core bibliometric database because it provides relatively complete citation indexing, cited references, journal information and institutional affiliations, making it suitable for co-citation, burst and collaboration network analyses. PubMed was used as a supplementary biomedical database to improve the coverage of clinical and medical literature. The complete search strategy is provided in Table 1.

**Table 1:** Search strategy.

Step	Pubmed	Wos
Search strategy	(“Child”[Mesh] OR “Adolescent”[Mesh] OR child*[Title/Abstract] OR adolescent*[Title/Abstract] OR pediatric*[Title/Abstract] OR paediatric*[Title/Abstract] OR youth[Title/Abstract]) AND (“Obesity”[Mesh] OR “Overweight”[Mesh] OR obes*[Title/Abstract] OR overweight[Title/Abstract]) AND (“metabolic phenotype”[Title/Abstract] OR “metabolic phenotypes”[Title/Abstract] OR “metabolically healthy obesity”[Title/Abstract] OR “metabolically unhealthy obesity”[Title/Abstract] OR “metabolic heterogeneity”[Title/Abstract] OR “phenotype transition”[Title/Abstract] OR “cardiometabolic risk”[Title/Abstract] OR “metabolic risk”[Title/Abstract])	TS = [(child* OR adolescent* OR pediatric* OR paediatric* OR youth) AND (obes* OR overweight) AND (“metabolic phenotype” OR “metabolically healthy obesity” OR “metabolically unhealthy obesity” OR “metabolic heterogeneity” OR “phenotype transition” OR “cardiometabolic risk” OR “metabolic risk”)]

### 2.3 Data processing

WoSCC records were exported as “Full Record and Cited References” in plain-text format, and PubMed records were exported in PubMed. A total of 4,393 records were retrieved from WoSCC and 2,046 from PubMed, yielding 6,439 records before deduplication. EndNote and the R bibliometrix package were used for data conversion, merging, duplicate removal and completeness checking. Duplicate records were identified using DOI, PMID, title, first author and publication year. After database merging and deduplication, 4,963 records were retained for bibliometric analysis, including 4,384 WoSCC records and 579 PubMed records. When the same publication appeared in both databases, the WoSCC version was retained because it contained more complete citation metadata. Because PubMed lacks complete cited-reference information, PubMed records were not used for co-citation, citation burst or citation-network analyses that depend on cited references.

**Figure 1:**
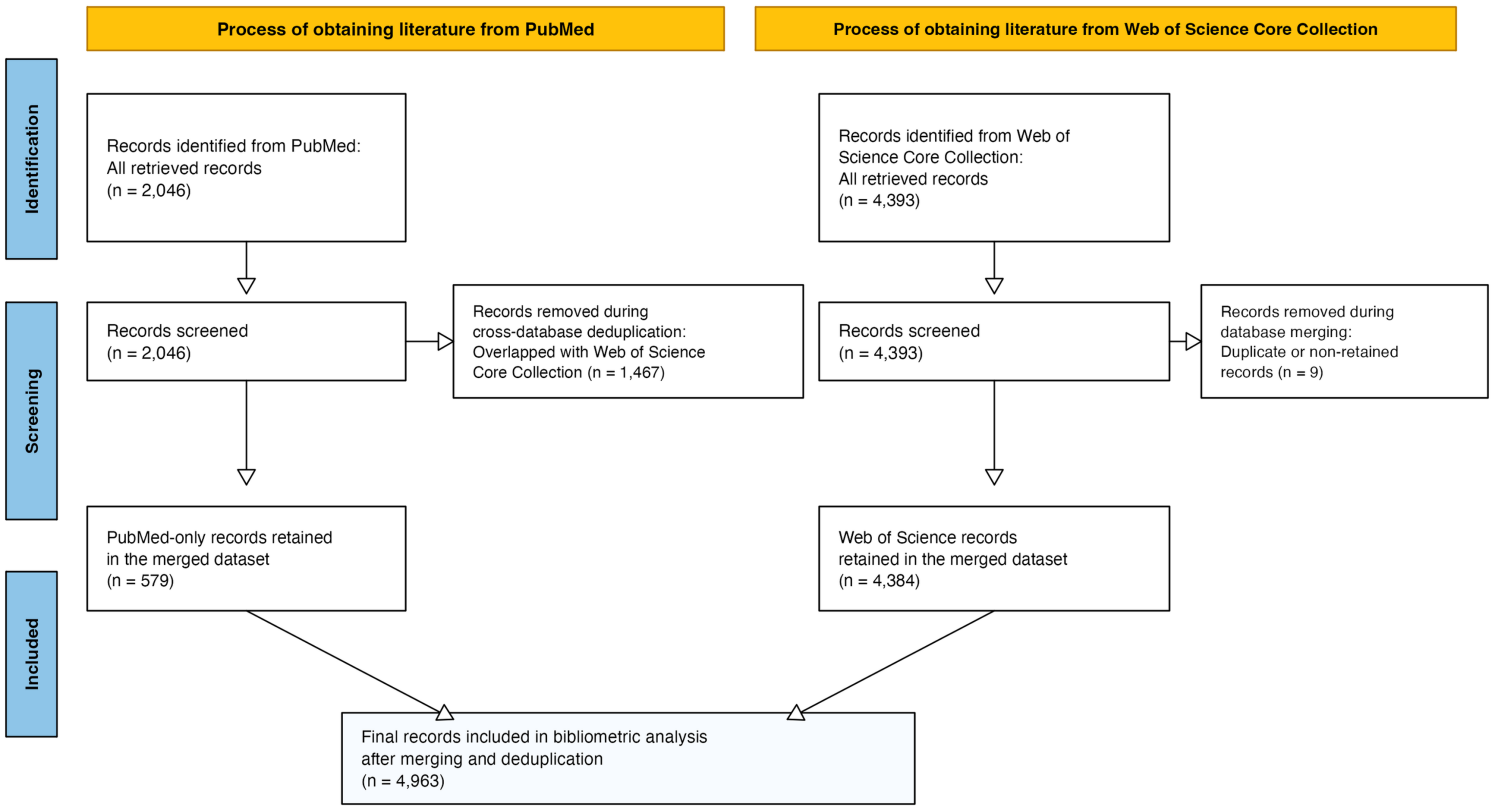
Flow chart of literature screening and bibliometric analysis workflow.

Before visualization, data standardization was performed. Author names were standardized by harmonizing initials, spelling variants and punctuation. Institutional affiliations were manually checked to merge different expressions of the same institution, including affiliated hospitals and abbreviated names. Keywords were standardized using a thesaurus file and manual checking. For example, “metabolically healthy obesity,” “metabolic healthy obesity” and “MHO” were unified, and “metabolically unhealthy obesity” and “MUO” were also merged (Figure 1).

### 2.4 Bibliometric and visualization analysis

CiteSpace 6.4. R1, VOSviewer 1.6.20, R software 4.5.2 and the bibliometrix package 5.4.1 were used for analysis (40–42). Bibliometrix was used for descriptive statistics and data-completeness checking. VOSviewer was used to analyze keyword co-occurrence, author collaboration, institutional collaboration and journal networks. CiteSpace was used to analyze country and institutional collaboration, author co-citation, reference co-citation, keyword clustering and burst terms.

The H-index was used to evaluate the sustained influence of authors or journals. Betweenness centrality was used to identify bridging nodes in the network. Total link strength was used to represent the overall strength of collaboration, co-occurrence or co-citation links between a node and other nodes. Full counting was applied in network analyses. In CiteSpace, the time slice was set to 1 year, and node types were selected according to the analysis objective, including country, institution, author, reference or keyword. Thresholds were set as Top *N* = 50 or g-index with *k* = 25. Cluster labels were generated using the log-likelihood ratio algorithm, and network pruning was performed using Pathfinder or pruning sliced networks. Keyword cluster quality was evaluated using modularity Q and mean silhouette S values; Q > 0.3 indicated a significant cluster structure, S > 0.5 indicated acceptable clustering and S > 0.7 indicated high reliability.

## 3 Results

### 3.1 Annual publication trends

**Figure 2:**
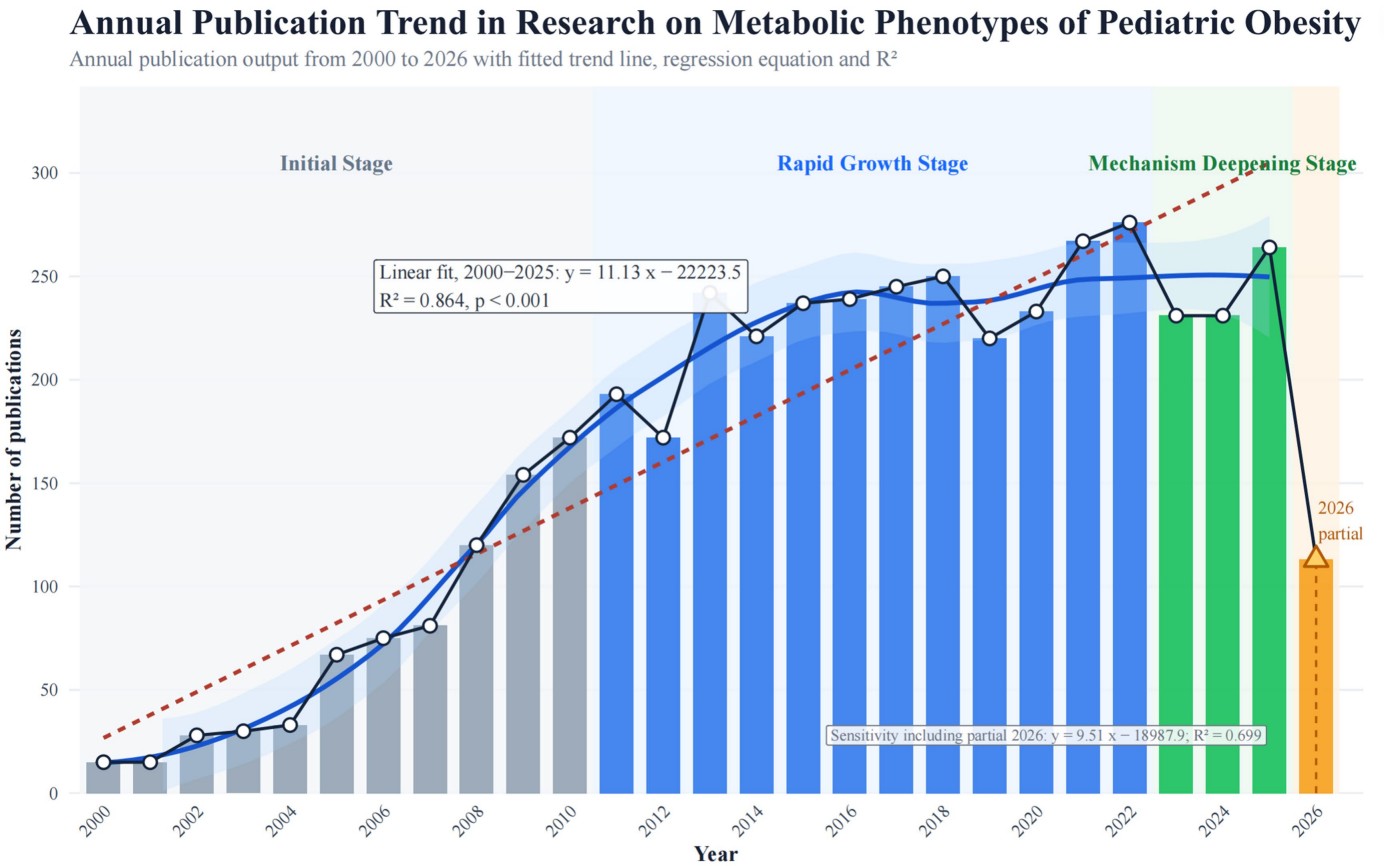
Annual publication volume of research on metabolic phenotypes in childhood and adolescent obesity.

The annual publication output on metabolic phenotypes in childhood and adolescent obesity showed an overall upward trend and could be divided into three broad stages. From 2000 to 2010, the field was in an initial phase, coinciding with the establishment of pediatric BMI cutoffs and metabolic syndrome criteria. From 2011 to 2022, publications increased markedly, likely reflecting the rising global burden of childhood obesity, guideline-driven prevention policies, increased funding for cardiometabolic risk research, and advances in body composition assessment, metabolomics and microbiome sequencing. From 2023 to 2025, output declined slightly from the peak but remained high, suggesting that the field has entered a consolidation phase focused on mechanisms, risk stratification and precision intervention. The fitted trend showed good explanatory power (*R*^2^ = 0.864, *p* < 0.001), supporting a robust long-term increase in research attention (Figure 2).

### 3.2 Analysis of countries, institutions and Core journals

**Figure 3:**
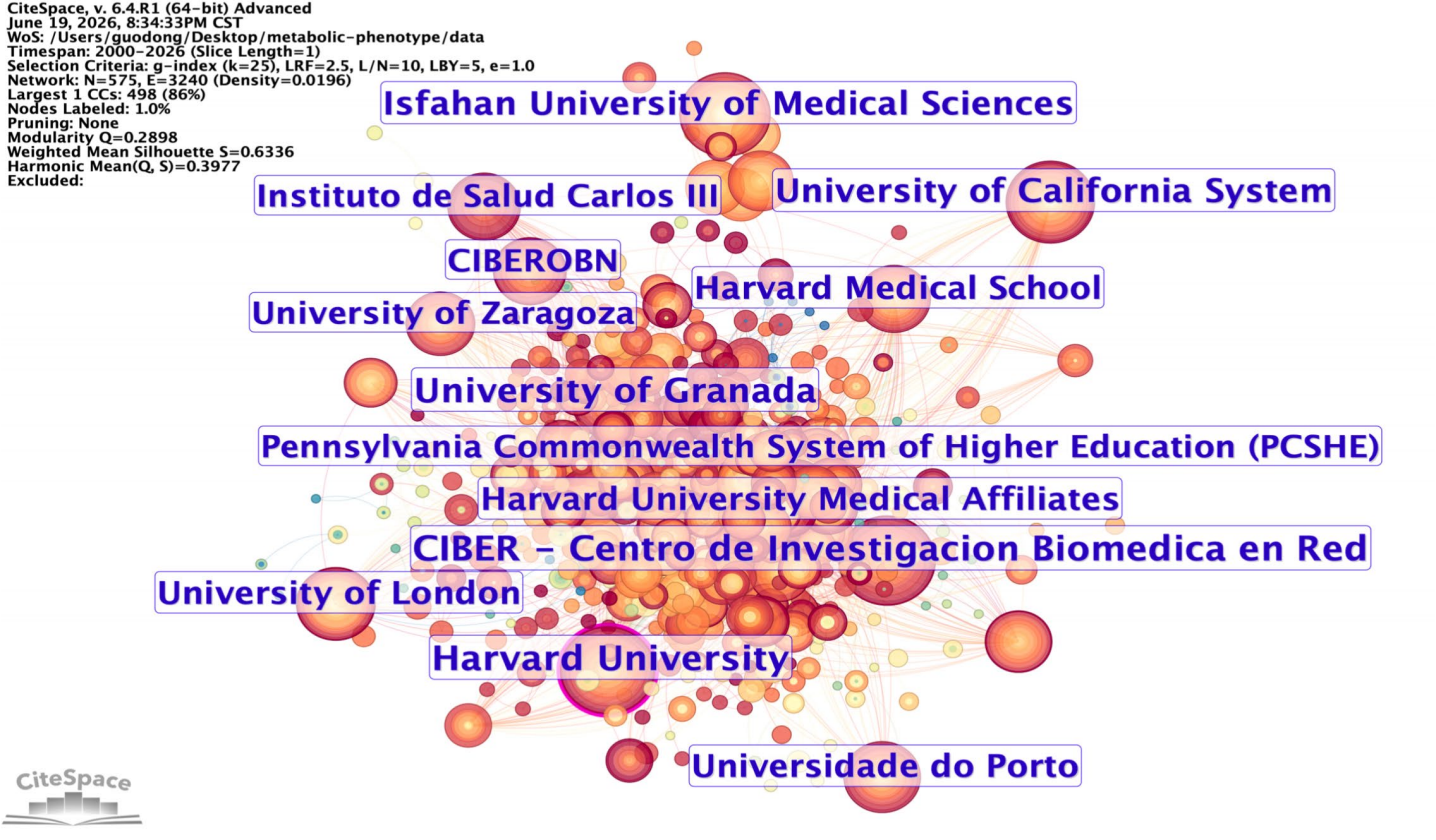
Institutional cooperation knowledge map in metabolic phenotype research on childhood and adolescent obesity.

The institutional collaboration network showed a broad but uneven pattern, with research activity concentrated mainly in North America, Europe and parts of Asia. Harvard University ranked first by publication output (*n* = 136), followed by CIBER - Centro de Investigación Biomédica en Red (*n* = 129) and the University of Granada (*n* = 106). This distribution suggests that the field has developed around several stable collaborative hubs, particularly in the United States and Spain, while participation from Asia and other regions is increasing but remains less central in the global collaboration network (Figure 3; Table 2).

**Table 2:** Top 10 institutions by publication output.

Rank	Institution	Count	Centrality
1	Harvard University	374	0.12
2	CIBER	203	0.07
3	University of Granada	106	0.07
4	Erasmus University Rotterdam	104	0.02
5	University of California System	99	0.05
6	Isfahan University of Medical Sciences	99	0.01
7	University of Colorado System	99	0.01
8	Universidade do Porto	84	0.01
9	Harvard Medical School	82	0.01
10	University of Zaragoza	76	0.01

**Figure 4:**
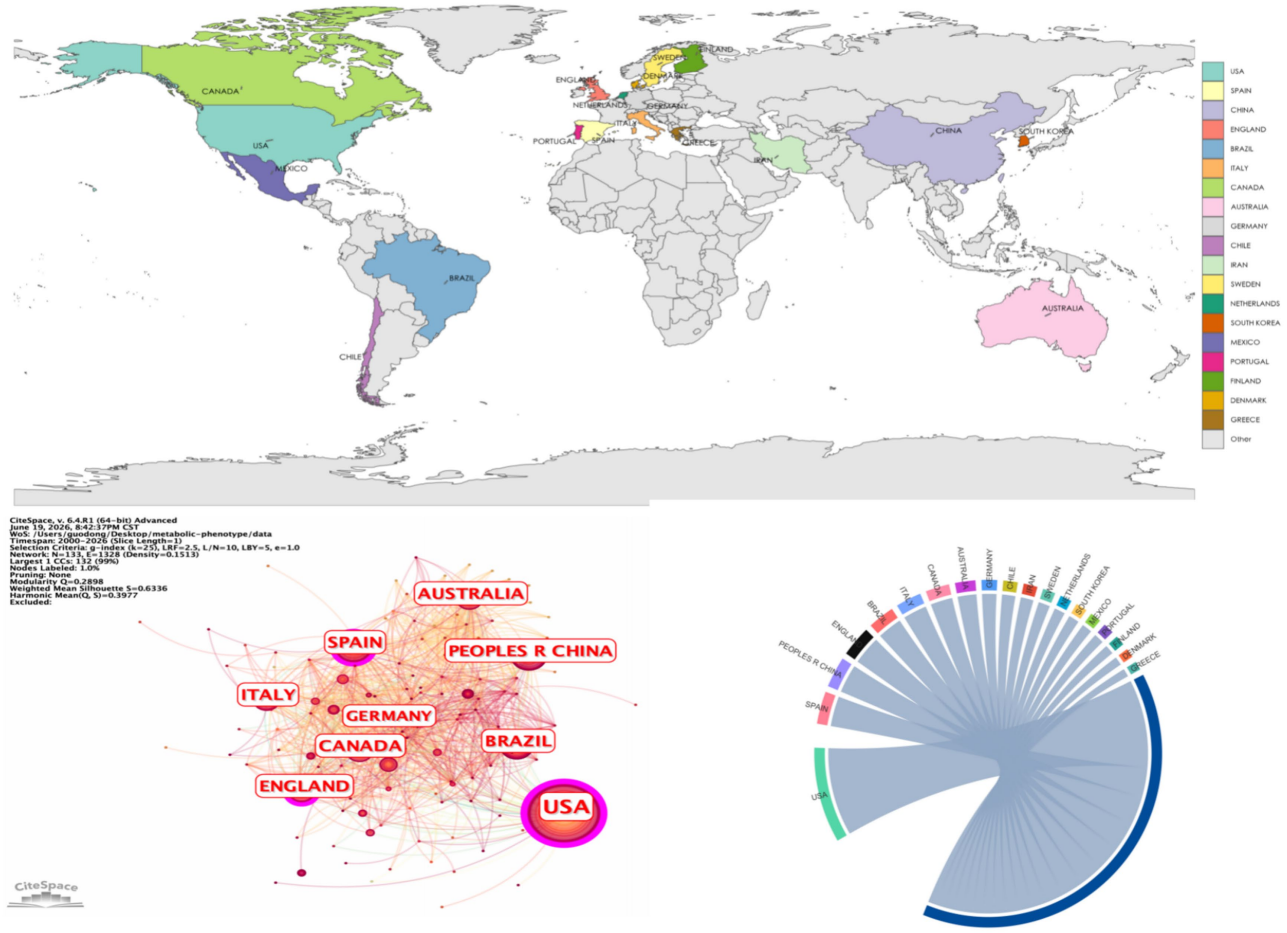
International cooperation network and global distribution map of metabolic phenotype research on childhood and adolescent obesity.

The country collaboration network showed broad but uneven international participation across North America, Europe, Asia and South America. The United States ranked first in publication output (*n* = 1,171) and centrality (0.26), suggesting that its leading role may be related to long-standing national health surveys, large pediatric cohorts, obesity-related funding priorities and early clinical guideline development. Spain (*n* = 399), China (*n* = 378) and England (*n* = 377) formed important secondary centers, likely reflecting strong public health research systems, increasing policy attention to childhood obesity and expanding investment in cardiometabolic disease prevention. Brazil, Italy, Canada and Australia further broadened the geographic distribution of the network. Overall, the field has developed a US-centered but increasingly international collaboration pattern, although cross-regional connectivity remains uneven (Figure 4; Table 3).

**Table 3:** Top 10 countries by publication output and centrality.

Rank	Country	Publications	Centrality	Year
1	United States	1,171	0.26	2001
2	Spain	399	0.11	2006
3	China	378	0.03	2008
4	England	377	0.17	2002
5	Brazil	326	0.02	2009
6	Italy	315	0.04	2002
7	Canada	292	0.09	2003
8	Australia	250	0.07	2007
9	Germany	191	0.07	2001
10	Chile	177	0.03	2007

**Figure 5:**
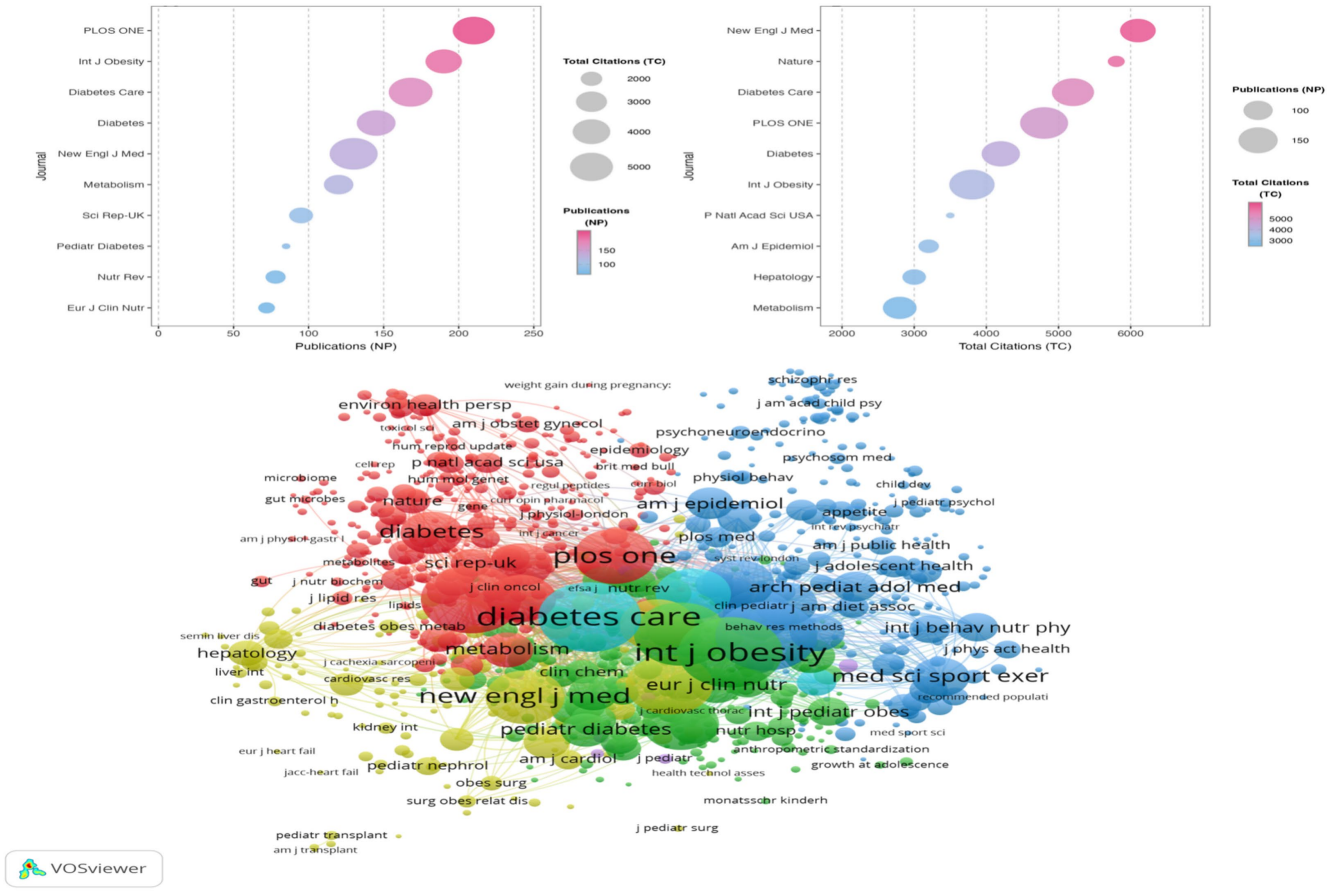
Core journal analysis diagram of metabolic phenotype research on childhood and adolescent obesity.

The journal distribution suggests that research on metabolic phenotypes in childhood and adolescent obesity is increasingly positioned at the intersection of nutrition, endocrinology, microbiology and general biomedical science. High-output journals such as Nutrients, Scientific Reports, Frontiers in Microbiology and Food and Function indicate that the field has expanded beyond traditional obesity research toward diet, metabolic regulation and microbiome-related mechanisms. In contrast, highly cited journals such as New England Journal of Medicine, Nature and Diabetes Care suggest that landmark evidence on metabolic syndrome, insulin resistance and cardiometabolic risk continues to shape the intellectual foundation of the field. The co-citation network further shows several connected thematic areas, including diabetes and metabolic mechanisms, pediatric obesity and nutrition, public health and behavioral intervention, and liver- or gut-related metabolism. Overall, these patterns indicate a transition from BMI-based description toward phenotype-specific interpretation, linking obesity heterogeneity with metabolic dysfunction, lifestyle exposure and precision prevention (Figure 5; Table 4).

**Table 4:** Top 10 productive journals in metabolic phenotype research on childhood and adolescent obesity.

Rank	Journal	Number of publications	Total citations	IF (2024)	JCR (2024)
1	Nutrients	195	5,463	5.0	Q1
2	Scientific Reports	108	3,965	3.9	Q1
3	Frontiers in Microbiology	106	2,946	4.5	Q1
4	Food and Function	102	2,653	5.4	Q1
5	International Journal of Molecular Sciences	100	3,628	4.9	Q1
6	PLoS One	87	5,835	2.6	Q2
7	Frontiers in Nutrition	86	1,260	5.1	Q1
8	Microorganisms	58	1,334	4.2	Q2
9	Gut Microbes	56	2,411	11.0	Q1
10	Journal of Agricultural and Food Chemistry	56	1,496	6.2	Q1

### 3.3 Co-cited references, burst references and co-cited authors

**Figure 6:**
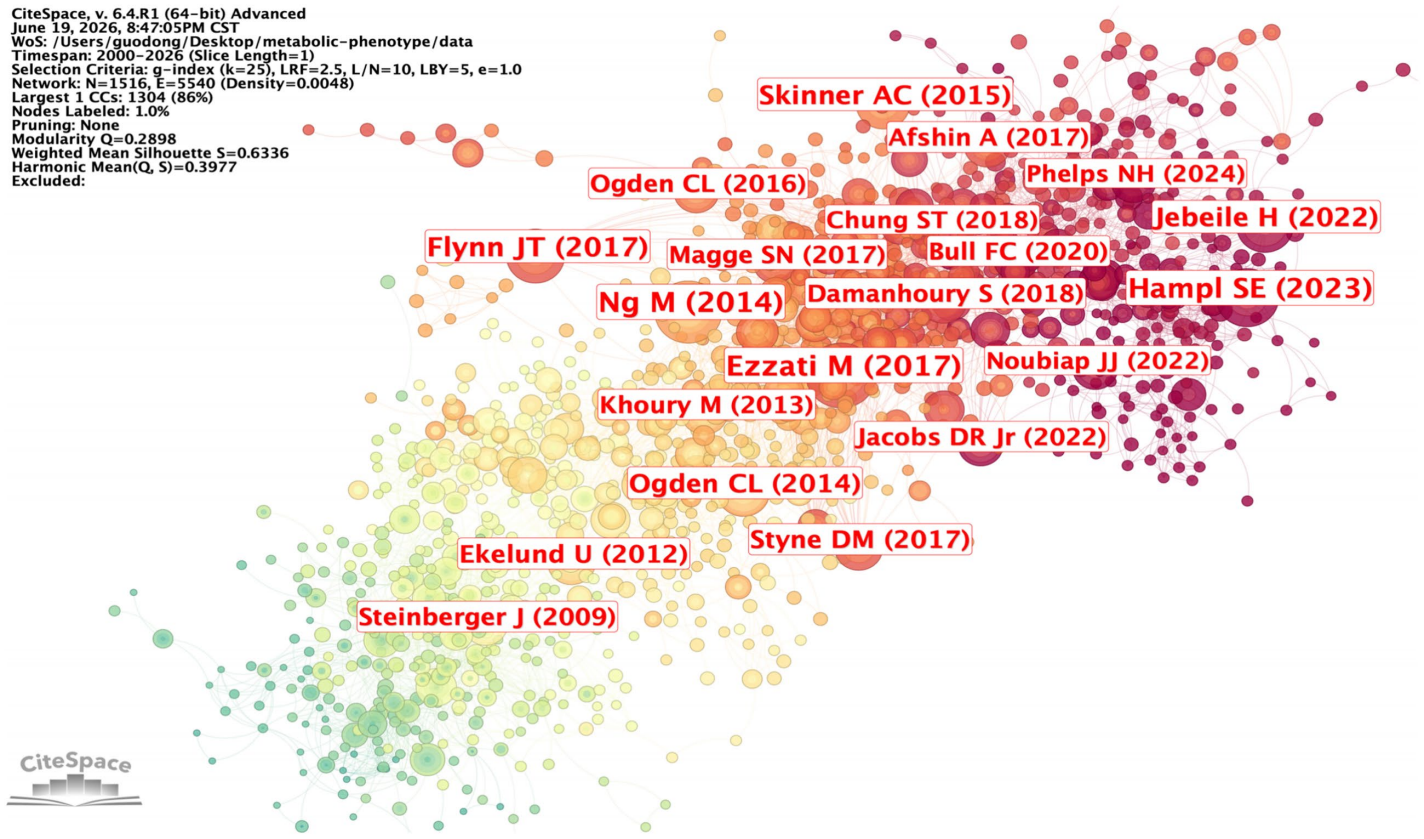
Co-cited reference knowledge map of metabolic phenotype research on childhood and adolescent obesity.

Reference co-citation analysis showed that the intellectual base of this field is concentrated in pediatric obesity trends, metabolic syndrome, glucose-lipid abnormalities and clinical management. The highly co-cited study by Weiss et al. (6) in the New England Journal of Medicine provided a key foundation by linking childhood obesity with metabolic syndrome and cardiometabolic risk. Studies by Ogden et al. (43) and Cook et al. (44) further anchored the field in obesity prevalence and adolescent metabolic syndrome phenotypes. Together, these references indicate that the field has evolved from describing pediatric obesity burden toward identifying metabolic risk patterns and guiding clinical assessment. More recent burst references on obesity guidelines, metabolic complications and lifestyle management suggest a further shift toward integrated management and phenotype-informed prevention (Figure 6).

**Figure 7:**
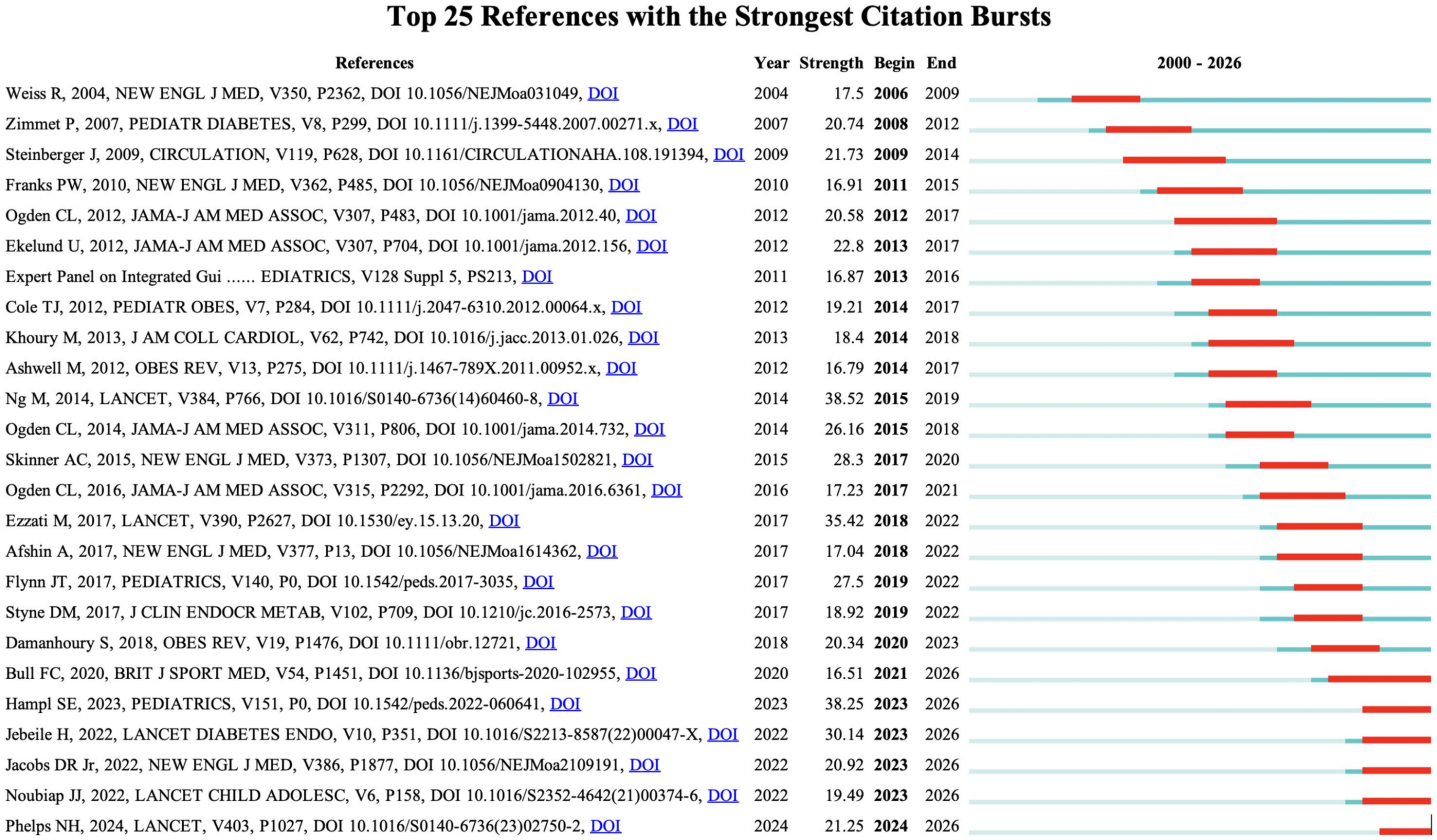
Top references with the strongest citation bursts in metabolic phenotype research on childhood and adolescent obesity.

Burst reference analysis further revealed a temporal shift in the knowledge focus of the field. Early burst references mainly addressed pediatric obesity, metabolic syndrome, glucose-lipid abnormalities, non-alcoholic fatty liver disease and clinical management guidelines, indicating that the field initially focused on defining obesity-related metabolic risks. The strongest burst was the 2004 article by Weiss et al. (6), followed by influential studies on obesity prevalence and adolescent metabolic syndrome (43, 44). More recent bursts, including the 2022 Lancet Diabetes and Endocrinology review and the 2023 Pediatrics clinical practice guideline, suggest that research attention is moving toward comprehensive assessment, standardized management and precision intervention for children and adolescents with obesity (8, 12) (Figure 7).

**Figure 8:**
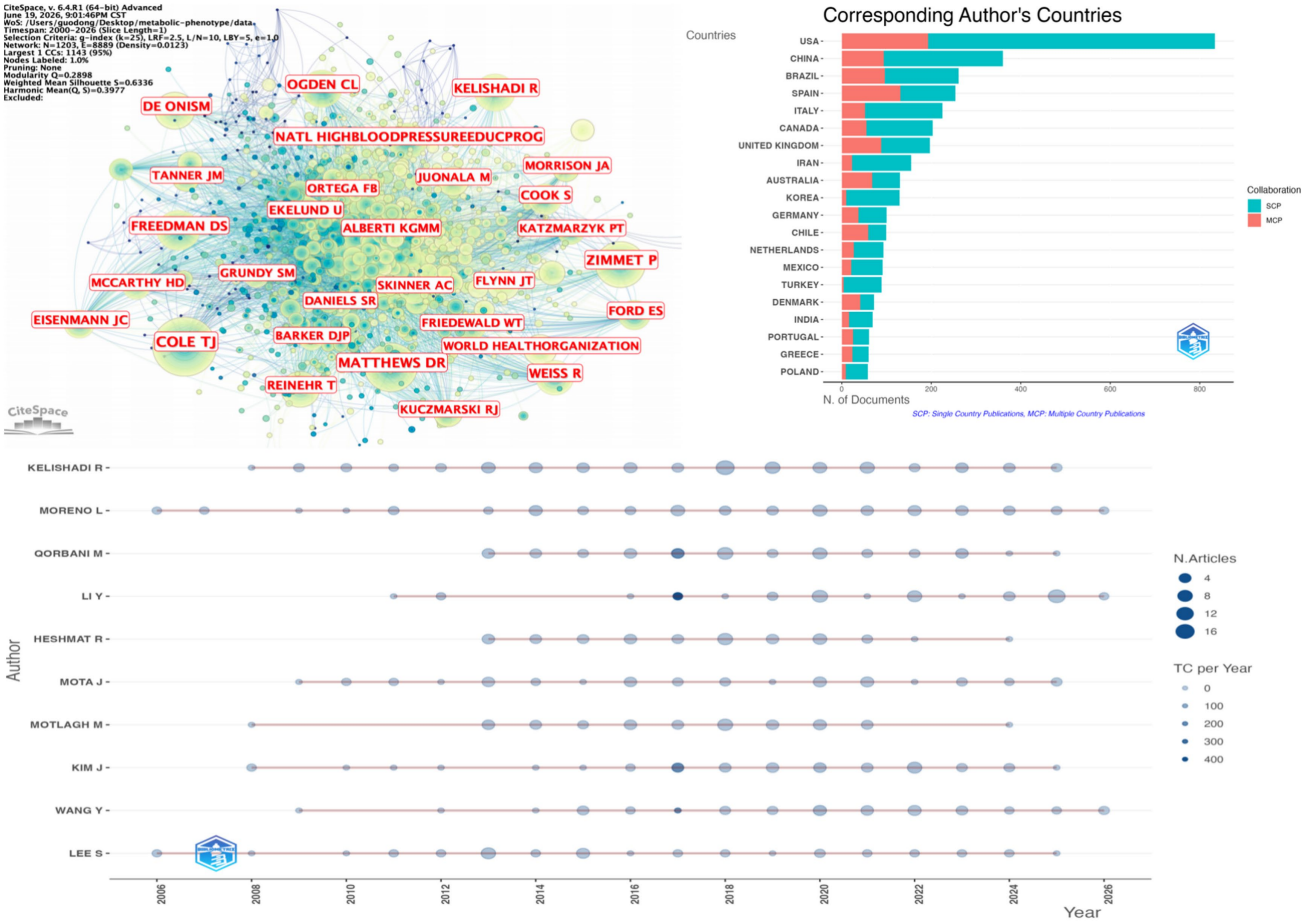
Co-cited author knowledge map in metabolic phenotype research on childhood and adolescent obesity.

Author co-citation analysis showed that the intellectual structure of this field is centered on growth standards, obesity epidemiology and metabolic risk assessment. Cole TJ ranked first in publication output and total citations (NP = 806; TC = 18,520), reflecting the enduring influence of international BMI cutoffs and standardized growth references. Matthews DR, Zimmet P, Freedman DS and Weiss R were also highly cited, likely because their work is closely linked to widely used methods for insulin resistance assessment, diabetes risk classification, metabolic syndrome definitions and pediatric obesity surveillance. The presence of Ogden CL, Kelishadi R, De Onis M, Reinehr T and Ekelund U further suggests that the field has been shaped by national health surveys, WHO growth standards, clinical guideline development and increasing attention to lifestyle-related cardiometabolic risk. Overall, the co-citation network indicates a gradual shift from anthropometric classification toward integrated cardiometabolic risk identification in children and adolescents with obesity (Figure 8; Table 5).

**Table 5:** Top 10 co-cited authors.

Rank	Author	Number of articles	H-index
1	Cole TJ	806	159
2	Matthews DR	543	108
3	Zimmet P	503	157
4	Freedman DS	461	92
5	Weiss R	405	68
6	Ogden CL	365	91
7	Kelishadi R	352	102
8	De Onis M	344	78
9	Reinehr T	336	87
10	Ekelund U	336	92

### 3.4 Sankey analysis

**Figure 9:**
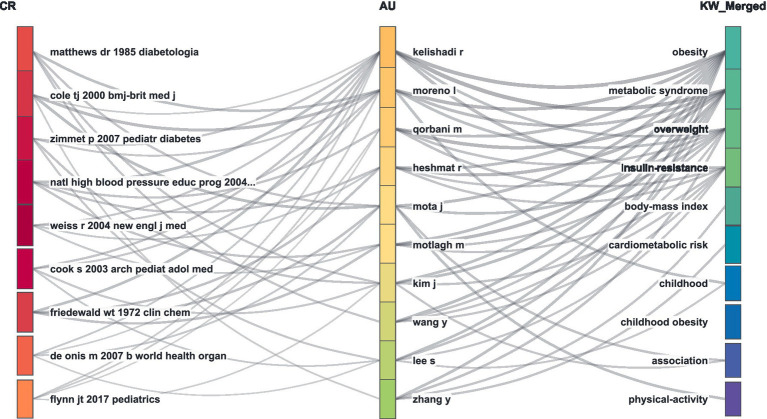
Sankey diagram linking cited references, authors and keywords in metabolic phenotype research on childhood and adolescent obesity.

The Sankey diagram linking cited references, authors and keywords showed a clear knowledge pathway from measurement standards and metabolic risk definitions to phenotype-oriented research. Foundational references on BMI cutoffs, insulin resistance, metabolic syndrome, blood pressure standards and pediatric obesity guidelines were connected with authors such as Kelishadi, Moreno, Qorbani and Heshmat. The main keywords, including obesity, metabolic syndrome, overweight, insulin resistance, body mass index, cardiometabolic risk and physical activity, suggest that the field has evolved from identifying childhood obesity and basic metabolic abnormalities toward integrated cardiometabolic risk stratification and lifestyle-related prevention (Figure 9).

### 3.5 Keyword clustering and burst terms

**Figure 10:**
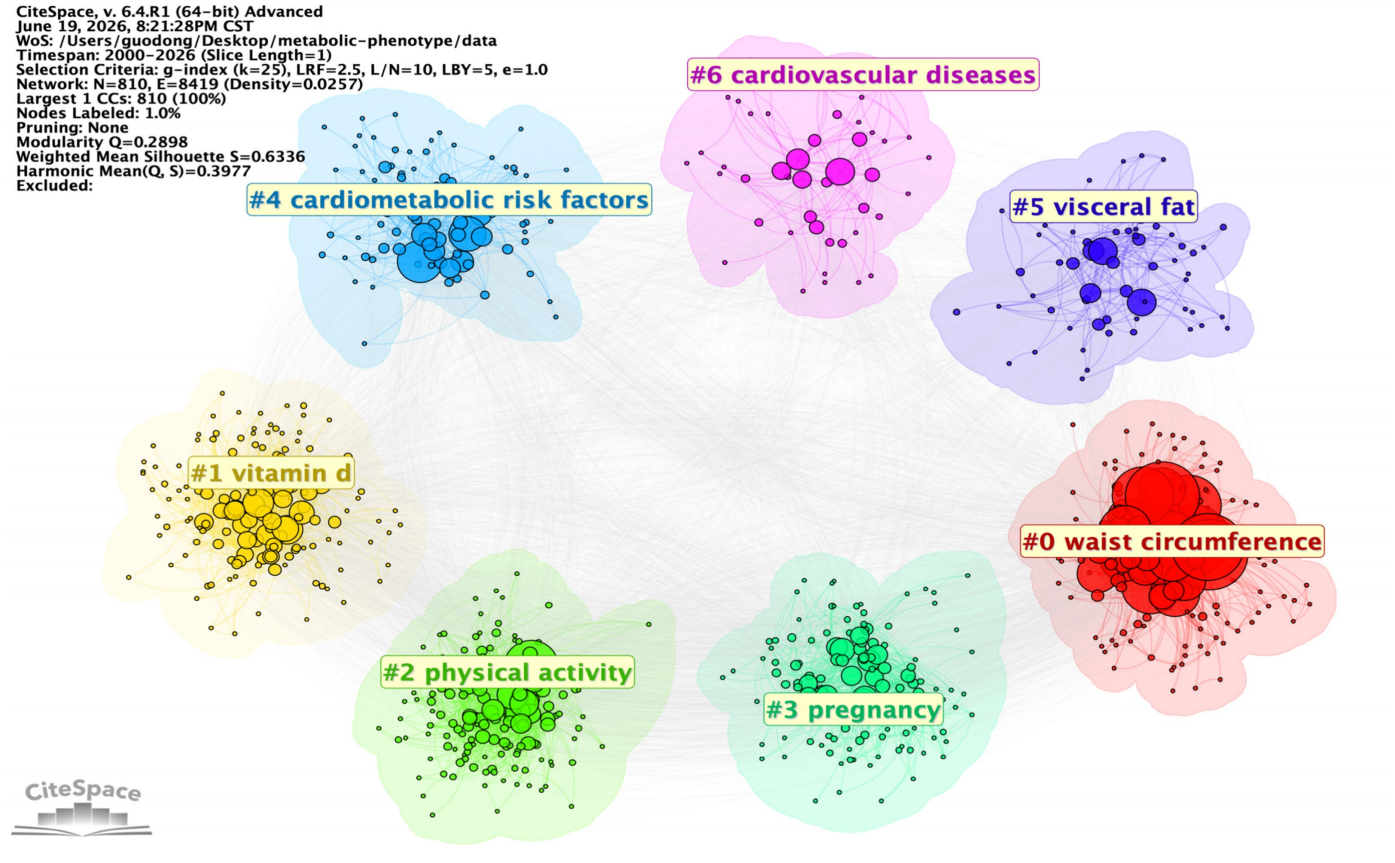
Keyword clustering map in metabolic phenotype research on childhood and adolescent obesity.

Keyword clustering generated a network with 867 nodes and 864 links with a density of 0.0023. The modularity *Q* value was 0.2738 and the mean silhouette S value was 0.6065 indicating acceptable cluster interpretability. Seven major clusters were identified: waist circumference vitamin D physical activity pregnancy cardiometabolic risk factors visceral fat and cardiovascular diseases. These clusters suggest that research hotspots have moved beyond general obesity classification toward more specific indicators of metabolic risk including central adiposity fat distribution micronutrient status lifestyle exposure and cardiometabolic outcomes. The prominence of waist circumference visceral fat and cardiometabolic risk factors indicates that phenotype research increasingly focuses on risk stratification rather than body weight alone (Figure 10).

**Figure 11:**
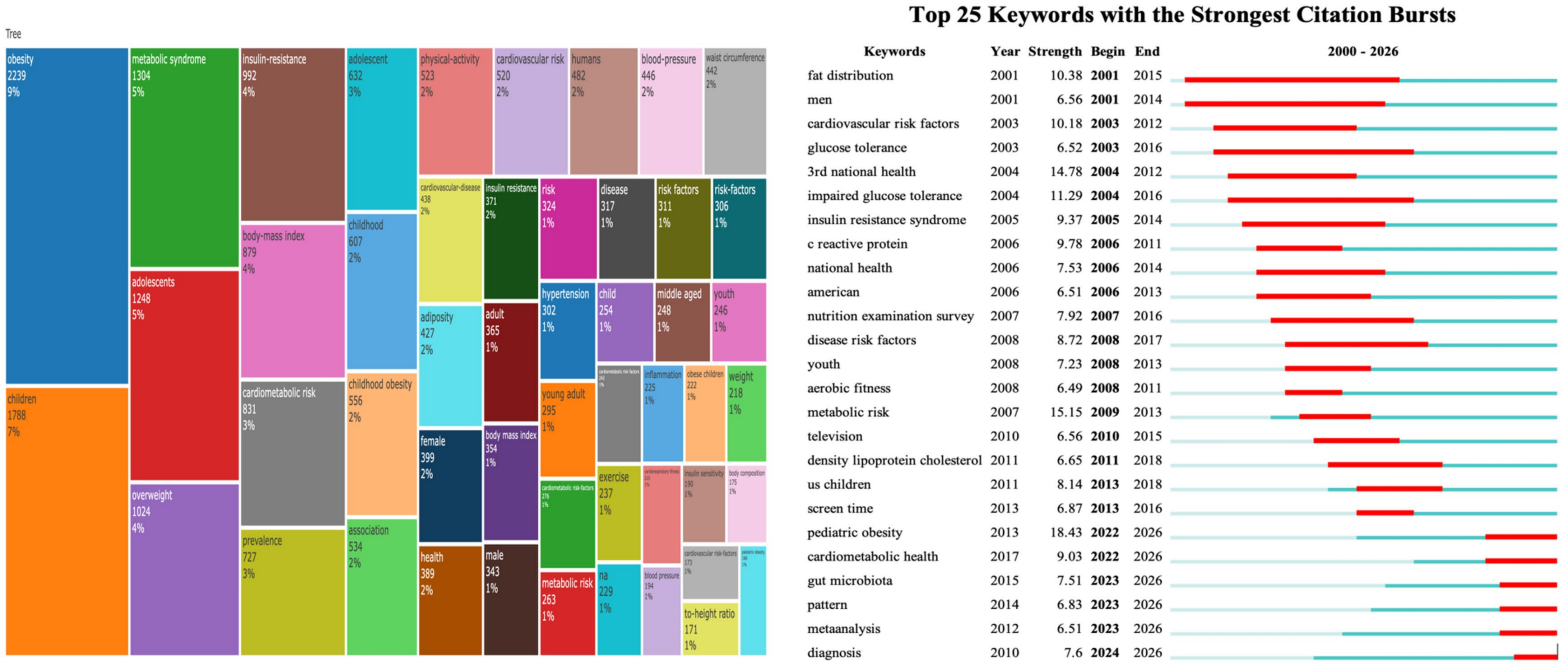
Keywords with the strongest citation bursts in metabolic phenotype research on childhood and adolescent obesity.

High-frequency keywords and burst terms indicated a temporal evolution from basic obesity classification to phenotype-specific risk interpretation. Stable keywords, including obesity, children, metabolic syndrome, adolescents, insulin resistance, overweight and body mass index, formed the long-term backbone of the field. Early burst terms such as fat distribution, glucose tolerance, insulin resistance syndrome and national health survey suggest that initial research focused on metabolic risk identification and population surveillance. These topics now appear less prominent as independent frontiers, serving more as foundational frameworks. Recent bursts, including pediatric obesity, cardiometabolic health, gut microbiota, pattern, meta-analysis and diagnosis, indicate emerging attention to microbiome-related mechanisms, diagnostic refinement and integrated cardiometabolic management. However, gaps remain in standardized phenotype definitions, longitudinal tracking of phenotype transitions and integration of lifestyle, microbiome and clinical markers into practical risk-stratification models (Figure 11).

## 4 Discussion

### 4.1 Bibliometric landscape and global context

This bibliometric analysis shows that research on metabolic phenotypes in childhood and adolescent obesity has evolved from BMI-based description toward metabolic risk stratification and phenotype-guided prevention. The increase in publications after 2011 likely reflects several converging factors: the global rise in pediatric obesity, the establishment of clinical guidelines, increased funding for cardiometabolic disease prevention, and advances in body composition assessment, omics technologies and microbiome research (8, 12–14). These changes allowed the field to move beyond whether childhood obesity is harmful and toward which children are metabolically vulnerable despite similar levels of adiposity.

The dominance of the United States and several European countries may be related to long-standing health survey systems, large pediatric cohorts, stronger research infrastructure and earlier clinical guideline development. However, the collaboration network also indicates regional imbalance. Countries with rapidly increasing childhood obesity burdens are not equally central in the network, which may limit understanding of ethnic, socioeconomic and environmental differences in metabolic phenotypes (54). Compared with previous bibliometric work focused mainly on childhood obesity and gut microbiota (42, 65), this study provides a broader phenotype-centered map linking obesity, insulin resistance, liver fat, physical activity, body composition and cardiometabolic risk.

Compared with previous bibliometric analyses on childhood obesity, gut microbiota and pediatric metabolic diseases, our findings are partly consistent in showing that obesity, insulin resistance, metabolic syndrome, gut microbiota and lifestyle intervention are major research hotspots (33, 44, 55, 64). However, the present study differs by focusing specifically on metabolic phenotypes in childhood and adolescent obesity rather than obesity burden or single mechanistic topics alone. This phenotype-centered perspective links BMI classification, cardiometabolic risk, liver fat, body composition, physical activity and gut microbiota within the same knowledge framework, thereby highlighting how the field is moving from general obesity description toward metabolic risk stratification and precision prevention.

### 4.2 From weight status to metabolic vulnerability

The co-citation, author and Sankey analyses suggest that the intellectual foundation of this field was built on BMI cutoffs, impaired glucose tolerance, insulin resistance and metabolic syndrome (5, 6, 33, 44). These early studies provided the framework for identifying children with excess weight and basic metabolic abnormalities. However, the current knowledge structure suggests a deeper conceptual transition: BMI is increasingly treated as an entry point rather than a sufficient explanation (52, 53, 55, 56).

This shift is biologically meaningful. Children with similar BMI may differ in visceral fat accumulation, hepatic fat deposition, insulin sensitivity, pubertal development, inflammatory burden and lifestyle exposure (50, 60, 62). These differences may explain why some children remain relatively metabolically preserved, whereas others show dyslipidemia, hypertension, impaired glucose metabolism or NAFLD. Therefore, the rise of terms such as waist circumference, visceral fat, cardiometabolic risk factors and body composition indicates that the field is moving toward markers that better capture adipose tissue dysfunction and ectopic fat-related risk than BMI alone (31, 35, 45, 46).

### 4.3 Thematic evolution and mechanistic implications

Keyword clustering and burst terms show three layers of thematic evolution. Stable themes include obesity children adolescents metabolic syndrome insulin resistance and BMI. These represent the long-term backbone of the field. Emerging themes include gut microbiota NAFLD physical activity cardiometabolic health diagnosis and phenotype-guided management. These topics suggest that recent research is increasingly concerned with mechanisms linking lifestyle exposure to metabolic deterioration (57, 58, 59, 61, 63).

Gut microbiota and liver fat are particularly important emerging nodes. The gut microbiota may reflect interactions among diet, inflammation, bile acid metabolism and host energy regulation, while NAFLD may serve as a visible marker of ectopic fat deposition and systemic metabolic dysfunction (31, 45, 47). Physical activity and dietary patterns also appear as sustained themes, suggesting that metabolic phenotypes are not fixed labels but dynamic states that may shift with growth, puberty and lifestyle context. In contrast, general obesity prevalence and basic BMI classification seem to be declining as independent frontiers, although they remain essential background frameworks (50, 51, 54, 58).

A key research gap is the lack of standardized pediatric phenotype definitions. Current studies often use different cutoffs for metabolic health, making comparison difficult. Another gap is limited longitudinal evidence. More prospective cohorts are needed to clarify whether metabolically healthy obesity is stable, transient or an early stage before metabolic deterioration. Intervention studies should also test whether children with different phenotypes respond differently to exercise, diet, sleep and behavioral strategies (64, 65).

## 5 Strengths and limitations

This study has several strengths. First, it focuses on metabolic phenotypes in childhood and adolescent obesity, a topic that links pediatric obesity, metabolic heterogeneity and cardiometabolic risk stratification. Second, by combining WoSCC-based bibliometric mapping with PubMed-assisted biomedical retrieval, this study improved literature coverage while avoiding the direct use of PubMed-only records for citation-dependent analyses when cited-reference metadata were incomplete. Third, the use of CiteSpace, VOSviewer and bibliometrix enabled cross-checking of publication trends, collaboration networks, co-cited references, author structures, keyword clusters and burst terms. Finally, this study goes beyond descriptive mapping by connecting bibliometric patterns with biological and clinical questions, including BMI limitations, phenotype transition, ectopic fat, insulin resistance, lifestyle exposure and precision prevention.

Several limitations should also be acknowledged. First, the findings should be interpreted as bibliometric associations and research trends rather than causal evidence. Publication growth, citation bursts and network centrality reflect research attention and knowledge connections, but they do not prove biological causality or clinical effectiveness. Second, database choice may influence the observed patterns. WoSCC provides structured citation metadata suitable for co-citation and burst analyses, whereas PubMed improves biomedical retrieval but contains incomplete cited-reference information. Third, the analysis was restricted to English-language articles and reviews, so non-English publications, regional journals and locally indexed studies may have been underrepresented. Finally, citation indicators are affected by publication year, journal visibility, field size and software parameters, and therefore should not be interpreted as direct measures of study quality.

## 6 Conclusion

Over the past 25 years, research on metabolic phenotypes in childhood and adolescent obesity has grown steadily and has shifted from external risk description toward MHO and MUO classification, mechanism exploration and phenotype-guided intervention. Future work should harmonize phenotype definitions, strengthen long-term follow-up and multicenter collaboration, and evaluate the differential effects of exercise, nutrition and lifestyle interventions across metabolic phenotypes. Such efforts may support a transition in pediatric obesity management from weight control alone toward precise prevention of cardiometabolic risk.

Overall, this study uniquely maps how pediatric obesity research has shifted from weight classification toward metabolic phenotype interpretation. It highlights the need for standardized definitions, multicenter cohorts, underrepresented regional data and mechanism-oriented intervention studies. These priorities may help future research and policy move from general obesity control toward early identification and prevention of cardiometabolic risk in metabolically vulnerable children.

## Data Availability

Publicly available datasets were analyzed in this study. This data can be found at: Web of Science Core Collection: https://www.webofscience.com/wos/woscc/summary/533fd7f8-905f-4d7b-8ca9-4de363adb76d-01bbff9bb7/relevance/1, PubMed: https://pubmed.ncbi.nlm.nih.gov/?term=%28%22Child%22%5BMesh%5D+OR+%22Adolescent%22%5BMesh%5D+OR+child*%5BTitle%2FAbstract%5D+OR+adolescent*%5BTitle%2FAbstract%5D+OR+pediatric*%5BTitle%2FAbstract%5D+OR+paediatric*%5BTitle%2FAbstract%5D+OR+youth%5BTitle%2FAbstract%5D+%29+AND+%28+%22Obesity%22%5BMesh%5D+OR+%22Overweight%22%5BMesh%5D+OR+obes*%5BTitle%2FAbstract%5D+OR+overweight%5BTitle%2FAbstract%5D+%29+AND+%28+%22metabolic+phenotype%22%5BTitle%2FAbstract%5D+OR+%22metabolic+phenotypes%22%5BTitle%2FAbstract%5D+OR+%22metabolically+healthy+obesity%22%5BTitle%2FAbstract%5D+OR+%22metabolically+unhealthy+obesity%22%5BTitle%2FAbstract%5D+OR+%22metabolic+heterogeneity%22%5BTitle%2FAbstract%5D+OR+%22phenotype+transition%22%5BTitle%2FAbstract%5D+OR+%22cardiometabolic+risk%22%5BTitle%2FAbstract%5D+OR+%22metabolic+risk%22%5BTitle%2FAbstract%5D+%29&filter=simsearch2.ffrft&filter=years.2000-2026&sort=fauth&size=200.
